# Electrophysiological Brain-Cardiac Coupling in Train Drivers during Monotonous Driving

**DOI:** 10.3390/ijerph18073741

**Published:** 2021-04-02

**Authors:** Ty Lees, Taryn Chalmers, David Burton, Eugene Zilberg, Thomas Penzel, Shail Lal, Sara Lal

**Affiliations:** 1 Edna Bennett Pierce Prevention Research Center, The Pennsylvania State University, 115 HHD Building, University Park, PA 16802, USA; tpl5365@psu.edu; 2 Neuroscience Research Unit, School of Life Sciences, University of Technology Sydney, P.O. Box 123, Broadway, NSW 2007, Australia; Taryn.Chalmers@uts.edu.au (T.C.); Shailendra.Lal@uts.edu.au (S.L.); 3 Compumedics Ltd., 30-40 Flockhart Street, Abbotsford, VIC 3067, Australia; dburton@compumedics.com.au (D.B.); ezilberg@compumedics.com.au (E.Z.); 4 Interdisciplinary Sleep Medicine Center, Charité Universitätsmedizin Berlin, 10117 Berlin, Germany; thomas.penzel@charite.de

**Keywords:** ECG, EEG, electroencephalography, fatigue, heart rate variability, monotony

## Abstract

Electrophysiological research has previously investigated monotony and the cardiac health of drivers independently; however, few studies have explored the association between the two. As such the present study aimed to examine the impact of monotonous train driving (indicated by electroencephalogram (EEG) activity) on an individual’s cardiac health as measured by heart rate variability (HRV). Sixty-three train drivers participated in the present study, and were required to complete a monotonous train driver simulator task. During this task, a 32 lead EEG and a three-lead electrocardiogram were recorded from each participant. In the present analysis, the low (LF) and high frequency (HF) HRV parameters were associated with delta (*p* < 0.05), beta (*p* = 0.03) and gamma (*p* < 0.001) frequency EEG variables. Further, total HRV was associated with gamma activity, while sympathovagal balance (i.e., LF:HF ratio) was best associated fronto-temporal delta activity (*p* = 0.02). HRV and EEG parameters appear to be coupled, with the parameters of the delta and gamma EEG frequency bands potentially being the most important to this coupling. These relationships provide insight into the impact of a monotonous task on the cardiac health of train drivers, and may also be indicative of strategies employed to combat fatigue or engage with the driving task.

## 1. Introduction

Professional driving is often characterised by lengthy periods of monotony, as drivers are frequently exposed to long stretches of relatively little stimulation and activity. Train driving is no exception, and is often considered more inherently monotonous than other driving industries because of the predetermined nature (with respect to time and destination/s) of the journey [1]. Indeed, the subjective experience of monotony has been associated with psychological distress and absence from work [2], cardiac risk factors [3], as well as reductions in task efficiency and performance [4]. Furthermore, train driving is often characterised by high workload, which results from rigid driving requirements and faced impediments, and has subsequently been linked to the development of driver fatigue. Driver fatigue has been associated with periods of hypovigilance [5,6], and increased likelihood of accidents [7,8,9]. This can have significant impact in train driving due to it inherently being a larger and heavier vehicle, which has a slower braking time and travels faster [10], and in the instance of passenger trains, the natural risk of carrying commuters.

Moreover, beyond the experience of driver fatigue, professional driving has been associated with a number of negative health consequences including stroke [11], coronary disease [12], ischemic heart disease [13], and myocardial infarction [14], as well as low physical activity [14] and other risk factors [15], which in turn have been associated with an increased overall disease burden [16] and risk of cardiovascular and metabolic diseases [16,17,18]. Additionally, it is important to note that health status has been shown to have an impact upon the safety of drivers [19], and consequently may contribute to accident incidence [20,21].

Numerous theories exist that aim at explaining this neural-cardiac link; for example, the psychophysiological coherence model postulates that specific cardiac activity patterns modulate the coherence between the cardiac and neural systems [22]. Whilst, neurovisceral integration theory [23,24] posits that cardiac vagal tone (as measured by heart rate variability; HRV) is associated with neural network functionality in affective-cognitive contexts. In all instances, electrophysiological research bridges the gap between driver fatigue and monotony and the possible impact on cardiac function of repeated/prolonged exposure to such conditions.

Previous research has primarily utilised spectral electroencephalography (EEG) to investigate monotonous driving and predict the onset and impact of driver fatigue [25,26,27,28,29,30]. Collectively, this research indicates that low frequency delta and theta brain activity both increase in fatigue and sleepiness [31,32,33,34], and are accompanied by a decrease in high frequency beta and gamma activity [35,36,37]. Heart rate variability has perhaps been more widely utilized with respect to its in electrophysiological research, having been used to investigate a wide variety of physiological conditions (e.g., diabetes and stroke), and psychological states (e.g., stress and fatigue) [38,39,40,41]. Broadly, HRV has been demonstrated to reflect the activity of the autonomic nervous system and its sympathetic and parasympathetic branches, [42] which control almost all visceral, vascular and metabolic functions [43]. Most importantly with respect to train driver health, reductions in HRV parameters have been shown to function as predictors for general mortality [44] and the development of cardiovascular risk factors including hypertension and obesity [45].

However despite the widespread use of both spectral EEG and HRV in electrophysiological and health research, studies investigating the association between these two approaches is often confined to acutely stressful or otherwise affective situations [46], and the investigation of such pairing in monotonous contexts is limited. Therefore, the present research aimed to investigate the relationship between brain activity and heart rate variability recorded during monotonous driving in train drivers. Based upon the aforementioned literature, e.g., [34,35,37] it was hypothesised that: (1) slow-wave EEG parameters (i.e., delta and theta activity) that are representative of monotony, fatigue and sleepiness would be associated with parasympathetic HRV, and; (2) fast-wave EEG parameters (i.e., alpha and beta activity) that are representative of alert and active states would be associated with sympathetic HRV.

## 2. Materials and Methods

### 2.1. Participants

Sixty-three train drivers (54 male, 9 female) aged between 24 and 69 years (average 38.56 ± 9.96 years) were recruited from the train driver population, and via Sydney Trains in Australia to participate in the current study. The Lifestyle Appraisal Questionnaire [47] was used to screen all recruited individuals for pre-existing medical conditions, as well as regular medication and other non-medical substance use that may have affected physiological measures. Additionally, pre-study blood pressure values of greater than 160 mmHg (systolic) and/or 100 mmHg (diastolic) were also used as an exclusion criteria, as these values are associated with an increased risk of cardiovascular events [48]. No recruited individual was excluded from participating in the present study. Finally, the study protocol was approved by the Human Research Ethics Committee, University of Technology Sydney. All participants provided written informed consent prior to participating.

### 2.2. Experimental Tasks and Procedures

Initially, participants were seated for a 5 min resting period, after which three blood pressure measurements using an automated sphygmomanometer (OMRON AIB) were obtained with a two minute rest between each reading as is recommended [49]; these values were then averaged to provide a pre-study blood pressure value for each participant. Following this, the participant was asked to completed the Lifestyle Appraisal questionnaire [47], which enabled the collection of participant demographic data. 

Upon completing the questionnaire, the electrophysiological monitors including a three lead electrocardiogram, and a 32 lead electroencephalogram were connected to the participant. ECG monitoring was completed using a FlexComp Infinity encoder (Thought Technology Ltd., Montreal, QC, Canada) supported by the BioGraph Infinity software package (Thought Technology Ltd., Canada). Prior to fixation, the adhesion sites for the ECG electrodes were cleaned with an alcohol wipe in order to remove surface debris and decrease any noise artefacts associated with an ECG signal. Following this, the ECG electrodes were placed with the active electrodes positioned at the intercostal space between the 4th and 5th ribs, two centimetres laterally from each side of the sternum, and the reference electrode as secured underneath the shoulder.

With respect to the EEG data capture, a 32 lead unipolar EEG montage with a sampling rate of 1000 Hz was recorded using a SynAmp2 amplifier (Compumedics Ltd., Abbotsford, VIC, Australia) supported by the Scan software (Version 4.3, Compumedics Ltd., Abbotsford, VIC, Australia). A 64 channel electrode QuikCap (Compumedics Ltd., Australia) was used; filled with Signa Gel (Parker Laboratories Inc, Fairfield, NJ, USA) at the following locations: Fp1, Fp2, F7, F3, Fz, F4, F8, FT7, FC3, FCz, FC4, FT8, T7, C3, Cz, C4, T8, TP7, CP3, CPz, CP4, TP8, P7, P3, Pz, P4, P8, O1, Oz, and O2. Further, the reference electrode was placed at the vertex, and the ground electrode at position AFz. Additionally, a bipolar vertical electrooculogram (EOG) was set up with one electrode above the left eye, and a second electrode below. 

Electrophysiological data collection was conducted during a 10-min resting baseline, as well as an active phase, which involved 10 min of train driving using the Trainz Classics (N3V Games, Helensvale, Australia) driving simulator program. During this active phase, participants engaged in a first-person train driving simulation task, in which they were required to correctly adhere to signals whilst undertaking straight-line track driving between stations and occasionally stopping at stations. After the active phase had been completed, the electrophysiological data collection was concluded. Finally, as an ethics requirement, three post-study blood pressure measures were again obtained and the experimental protocol was concluded.

### 2.3. Electrophysiological Data Processing

Prior to statistical analysis, both sets of raw electrophysiological signals (ECG and EEG) were pre-processed, so as to obtain the relevant and summarizing variables in the time and frequency domains.

#### 2.3.1. Heart Rate Variability

The recorded ECG data was processed utilising the Kubios HRV Premium software (Version 3.1.0, Kubios Oy, Kuopio, Finland) to derive the activity for the following HRV frequency bands: low frequency (LF; 0.04–0.15 Hz) and high frequency (HF; 0.15–0.4 Hz). The automatic artefact correction process built into the Kubios software [50], as well as the Smoothn Priors method [51] of trend component rejection were utilised to correct any artefacts and ectopic beats present in the raw ECG recordings. 

All variables were derived from approximately 10 min of ECG [52], utilising Welch’s periodogram method [53] applied to a 300 s window with a 50% overlap. Further, it should be noted that for each of these variables raw power units (ms^2^) as well as natural log transformed (ln) and normalised units (n.u.) were utilised. Additionally, the ratio between LF and HF (LF/HF) as well as overall HRV power or total power (TPow) was also computed. 

#### 2.3.2. Electroencephalography Data

The raw EEG data sets were filtered using a Butterworth IIR bandpass filter set at 0.5 and 50 Hz, which was followed by a Hanning window. The Aligned-artefact average procedure utilising vertical EOG data [54,55] was then applied to minimise any eye movement artefacts, and any muscle artefacts and movement artefacts were also removed as well. After pre-processing, both the baseline and active recordings were sectioned into approximately 300 two second epochs, and the EEG activity in the delta (0.5–4 Hz), theta (4–8 Hz), alpha (8–13 Hz), beta (13–35 Hz) and gamma (35–50 Hz) frequency bands [56] was calculated via Periodogram power spectral density estimate. The epoch values of each recording were scanned for outliers, which were removed using a modified z-score statistic [57] greater than or equal to 5, which was calculated using the following equations:

(1) $$Z=\frac{X-\tilde{x}}{\mathrm{MAD}}$$

where: X = Epoch value; $\tilde{x}$ = Median value; MAD = Median Absolute Deviation.

Median Absolute deviation can be calculated using the following equation:

(2) $$\mathrm{MAD}=\tilde{x}(|X_{i}-\tilde{x}(X_{i})|)$$

where: $\tilde{x}$ = Median; Xi = Epoch value.

After the removal of epoch outliers, the activity values of each epoch were averaged to derive a single value in each frequency band for each electrode location. Additionally, all activity values in each EEG frequency band were also averaged, providing an overall average EEG activity value. Finally, after all EEG data had been collated, outlying values were again removed using the modified Z-score statistic previously applied to the epoch values; however, the threshold at which values were removed in this instance was set at a z statistic greater than or equal to 10 [57].

### 2.4. Data Analysis

The present statistical analysis was conducted using STATISTICA (Version 10, 2011, Statsoft Inc., Tulsa, OK, USA) with statistical significance set at *p* < 0.05. Partial Pearson’s correlation analysis controlling for participant age and body mass index (BMI) was used to determine any associations between EEG variables and HRV variables. If a significant association between three or more independent EEG variables and a single dependent HRV variable were identified via correlation analysis, multiple general linear regression analysis was utilised to determine the strongest individual predictor.

## 3. Results

Log-transformed low frequency HRV (LFln) was significantly and negatively correlated to frontal pole theta and alpha activity as well as fronto-temporal beta activity (Table 1). Furthermore, positive associations were found between LFln and occipital delta and gamma activity, as well as parietal gamma activity (Table 1).

A forward stepwise general linear regression analysis informed by the significant correlations was performed to examine the relationship between LFln HRV and EEG parameters (Table 2). Of the 6 originally entered variables, the regression analysis for LFln retained 3 (O1 delta activity, FT8 beta activity, and O1 gamma activity), and had an overall significance of *p* = 0.01. Together, these 3 variables explained 52.4% of the variance in LFln (F = 5.14; df = 3; *p* = 0.01; R = 0.72; R^2^ = 0.52; AR^2^ = 0.42). Furthermore, FT8 beta activity also presented as independently significant predictors of LFln (*p* = 0.03) in the regression.

When examining normalised LF HRV, the present analysis determined positive correlations for central delta activity, and frontal pole alpha activity (Table 3). Furthermore, parietal beta and gamma activity, and occipital gamma activity were significantly and negatively correlated with the LFn.u. parameter.

To further examine the relationship between LFn.u. HRV and EEG parameters, a forward stepwise general linear regression analysis informed by the significant correlations was performed (Table 4). Of the variables entered into the regression analysis, two were retained (C3 delta activity and O1 gamma activity) with an overall significance of *p* < 0.001. Together, these 2 variables explained 57% of the variance in LFn.u. (F = 13.11; df = 2; *p* < 0.001; R = 0.75; R^2^ = 0.57; AR^2^ = 0.52). Furthermore, both variables also presented as independently significant predictors of LFn.u. (*p* < 0.05 and *p* < 0.001, respectively).

With reference to high frequency HRV, HFln was significantly negatively correlated to fronto-temporal and central delta activity, as well as frontal pole alpha and fronto-temporal beta activity. Furthermore, parietal and occipital gamma activity were positively correlated to the HFln parameter (Table 5).

The associations of log-transformed HF HRV with EEG parameters were further investigated using a forward stepwise general linear regression analysis informed by the significant correlations (Table 6). Of the 7 originally entered variables, the regression analysis for HFln retained 3 (C3 delta activity, FT8 beta activity and O1 gamma activity), and had an overall significance of *p* < 0.001. Together, these 3 variables explained 62% of the variance in HFln (F = 9.14; df = 3; *p* < 0.001; R = 0.79; R^2^ = 0.62; AR^2^ = 0.55). Furthermore, O1 gamma activity also presented as an independently significant predictor of HFln. (*p* < 0.001).

Similar results were determined for normalised HF HRV, which was also significantly and negatively correlated to central delta activity and frontal pole alpha activity (Table 7). Moreover, parietal beta and gamma activity, and occipital gamma activity were positively correlated with HFn.u.

Similarly, a forward stepwise general linear regression analysis investigated the associations between normalised HF HRV with EEG parameters (Table 8). Of the 6 originally entered variables, the regression analysis for HFn.u. retained 2 (C3 delta activity, and O1 gamma activity), and had an overall significance of *p* < 0.001. Together, these variables explained 56% of the variance in HFn.u. (F = 12.97; df = 2; *p* < 0.001; R = 0.75; R^2^ = 0.56; AR^2^ = 0.52). Furthermore, both variables also presented as independently significant predictors of HFn.u. (*p* =< 0.05 and *p* < 0.001, respectively).

Total HRV power was negatively correlated with frontal pole delta, theta and alpha activity, as well as fronto-temporal beta activity (Table 9). Furthermore, positive associations between TPow and parietal and occipital gamma activity were also determined.

Log-transformed total heart rate variability power and its association with EEG parameters was also further investigated using forward stepwise general linear regression analysis (Table 10). Of the 6 originally entered variables, the regression analysis for TPowln retained 2 (P4 and O1 gamma activity), and had an overall significance of *p* < 0.001. Together, these 2 variables explained 65% of the variance in TPowln (F = 15.98; df = 2; *p* < 0.001; R = 0.81; R^2^ = 0.65; AR^2^ = 0.61). Furthermore, O1 gamma activity also presented as an independently significant predictor of TPln. (*p* < 0.001).

Finally, the present analysis determined that the LF/HF ratio was significantly and positively correlated to fronto-temporal delta and theta activity, as well as fronto-central theta activity, frontal pole alpha activity, centro-parietal beta activity, and fronto-central and centro-parietal gamma activity (Table 11).

The associations between the LF/HF ratio and EEG parameters were further investigated using a forward stepwise general linear regression analysis informed by the significant correlations (Table 12). Of the 8 originally entered variables, the regression analysis retained 2 (FT8 delta activity, and FC4 theta activity), and had an overall significance of *p* < 0.001. Together, these 2 variables explained 61% of the variance in LF/HF (F = 13.07; df = 2; *p* < 0.001; R = 0.78; R^2^ = 0.61; AR^2^ = 0.56). Furthermore, FT8 delta activity also presented as an independently significant predictor of LF/HF (*p* = 0.02).

## 4. Discussion

The present study aimed to investigate the relationship between brain activity and HRV recorded in train drivers during a monotonous train driving task. Associations between independent frequency domain HRV parameters (LF and HF) and both low and high frequency EEG variables were observed. More specifically, increased LF HRV was associated with increased delta activity, reduced beta activity, and a location dependent increase or decrease in gamma activity. Increased HF HRV was associated with reductions in delta and beta activity, as well as increased gamma activity. Interestingly, sympathovagal balance (LF:HF) was only correlated with low frequency EEG parameters, being positively associated with fronto-temporal delta and fronto-central theta activity.

### 4.1. Low and High Frequency Heart Rate Variability

Within frequency domain HRV research, the LF parameter has typically been considered to be in part representative of sympathetic nervous system activity, particularly once it is transformed or normalised [52]. Presently, increased sympathetic activity (as indexed by LF HRV) was associated with increased central and occipital delta activity, reduced fronto-temporal and parietal beta activity, and a potential gamma oscillation, whereby both an increase and decrease in occipital gamma was found. 

Previous electrophysiological investigations of fatigue and sleepiness have demonstrated that delta activity increases [32,33,34], and high frequency beta and gamma activity decreases [35,36,37] during these states. When considered in this context, it stands to reason that the observed correlation and increase in low frequency EEG, i.e., delta activity and reduction in high frequency EEG (i.e., beta and gamma) activity may be indicative of the train drivers actively experiencing monotony-related fatigue [31]. Alternatively or perhaps supplementarily, past research has noted that professional drivers, including train drivers, engage and utilise various tactics to actively combat and compensate for fatigue, most of which are typically associated with increases in the amount of consciously applied effort [58,59]. Activation of the sympathetic nervous system fundamentally represents the fight or flight response and it’s underlying mechanics, whereby an individual actively either engages (i.e., fights) or disengages (i.e., flights) with a distressing stimuli [60]; and so, it could be postulated that these combative efforts may be facilitated by or at the very least associated with increases in sympathetic activity. Thusly, it could be suggested that the neural-cardiac coupling between an increase in LF HRV and an associated increase in slow-wave and decrease in fast-wave EEG activity may electrophysiologically represent attempts to more actively engage with the driving task, or the combative responses that train drivers often employ in an attempt to overcome experienced monotony/fatigue. However, it should be noted that this is speculative, and future studies may wish to actively image both the task engagement and fatigue ‘countermeasures’ used by train drivers to explicitly examine these effects.

Continuing the focus on LF HRV, previous research has associated elevated LF HRV to have been linked with the increased presence of cardiac risk factors and cardiac disease [61,62]. Furthermore, physiologically constant and prolonged sympathetic activity has been show to place potentially damaging pressure on cardiac health [63]. As such, it could be inferred that the experience of monotony and fatigue is increasing the cardiac risk and threatening the cardiac health of these train drivers. A particularly poignant inference, as professional driving as an industry itself has also been previously associated with increased cardiac risk and chance of developing cardiac disease [12,13,14]. Further, it is possible that these bodies of literature are describing the same effect, i.e., the increase in LF HRV and cardiac risk, is the same industry specific increase in cardiac risk. However, it may also be possible that the observed cardiac effects could compound and/or potentially exacerbate one another, and future research would do well to consider separating the two effects (i.e., the cardiac impact of monotony, and the cardiac impact of professional driving) to explicitly test this. Nonetheless, not only do the present associations between LF HRV and EEG provide insight into neural-cardiac coupling during monotony and the potential cardiac risk of such conditions, but also demonstrates the potential to form a basis from which such electrophysiological monitoring could be applied towards the active assessment of driver health in situ, and in monotonous environments.

With respect to HF HRV, it is thought to be the complement to LF HRV and so is considered to be representative of parasympathetic nervous system activity [38]. In the present analysis increased parasympathetic activity (i.e., HF HRV) was associated with reductions in central delta and fronto-temporal beta activity, and increases in occipital gamma activity. Interestingly, the traditional “rest and digest” characteristics that are attributed to parasympathetic activity would suggest that rather than the aforementioned observed associations, one would expect to see an increase in delta activity and a reduction in high frequency EEG activity. Furthermore, these results are close to the inverse (i.e., decreased slow-wave activity and increased fast-wave activity) of the correlations that were observed when examining the neural coupling of LF HRV, potentially providing a look at the push-pull interaction of the arms of the autonomic nervous system, and perhaps an alternate parasympathetic view of monotony characterized by reductions in delta and beta activity combined with potential gamma bursts. As to what these correlations may represent, perhaps given the separate nature of LF and HF with respect to autonomic function, this second set of correlations reflects monotony-related task disengagement, which may be driving the previously purported increase in conscious effort. Alternatively, it is quite possible that these correlations simply represent some unmonitored cognitive component of the simulator task like the driving events (e.g., track signal changes), in particular the increase in gamma activity, which has often been correlated with cognitive events [64]. On that note, future research investigating not only neural-cardiac coupling but monotony and fatigue more broadly should consider eye-tracking or other techniques that enable the monitoring of participant attention to visualise both disengagement and any increases is cognitive effort, as well as the flagging of all events (e.g., track signals, station stops, etc.) that participants experience.

### 4.2. Total Heart Rate Variability and Sympathovagal Balance

Total heart rate variability was also considered in the present analysis and was most strongly associated with parietal and occipital gamma activity, whereby an increase in total HRV power would be accompanied by increases in these high frequency EEG parameters. Additionally, the present analysis also strongly associated increases in sympathovagal balance (as represented by the LF/HF parameter) with increases in fronto-temporal delta and fronto-central theta activity. Given the observed correlation analyses for both the LF and HF HRV parameters, it seems reasonable to suggest that the series of correlations between TP and LF/HF HRV and EEG parameters are most likely attributable to the influence of LF HRV, and could be similarly indicative of a state of sympathetic dominance, whereby an individual reports LF HRV greater than their HF HRV. 

Within this context, it again should be noted that sympathetic dominant HRV has been associated with cardiac risk [62], and so the results observed for total HRV power and sympathovagal balance not only provide further support for neural-cardiac coupling experienced during monotony, but in-turn also provide some insight into the cardiac health of train drivers, as well as potentially that of other workers and operators in other industries that also frequently experience monotonous or otherwise stressful work settings.

### 4.3. Limitations & Future Directions

As with all research, the present study is not without its limitations. The present protocol utilised an approximately 10-min monotonous train driving task to collect and examine HRV and brain activity. Whilst the task itself is designed to be inherently monotonous, it is relatively short, and a lengthier driving task [9,29] may provide greater insight into monotony and its cardiac implications. This could be particularly important if the electrophysiological parameters capture any potential phasic shifts related to individual awareness of experiencing monotony and fatigue, and/or any combative actions and/or increases in conscious effort made by drivers to counter their experience of fatigue [59].

In relation to the electrophysiological parameters, the present work utilised a relatively small 32-lead monopolar EEG montage and future research may wish to expand on the electrode montage to provide greater nuance or open up the possibility of using source-localisation approaches. Further, expanding the derived variables of interest from channel specific spectra to include synchronicity, coherence [65], interaction parameters, i.e., power spectral ratios, and/or other non-traditional EEG [66], may allow for a better representation of monotony and fatigue [26,67], and hold additional insight into the cardiac impact of this experience [40]. 

Similarly, the present experiment confined the derived HRV parameters to only the traditional frequency domain variables, and an expansion into examining time-domain and other non-linear HRV parameters, such as entropy values, may also provide additional insight into brain-cardiac coupling in monotony and its implications in train drivers. Additionally, beyond the specific parameters themselves, auto-regressive HRV generation/pre-processing techniques also warrant consideration in future research. Further, with respect to the HRV data, the two datasets (i.e., log-transformed and normalised) differently represent the activity of the autonomic nervous system. More specifically, log-transformed data broadly comments on autonomic data, whilst normalised HRV focuses more specifically on the individual autonomic arms. To that end, while this distinction has no bearing on the present work, future studies may wish to engage with this difference more actively.

Moving forward, with respect to analytical options, future research should consider the value of both repeated measures designs and the application of data normalization techniques (e.g., independent/principle component analysis) in their handling of electrophysiological data as it is possible that such approaches may provide additional insight into the link between the two physiological measures. Moreover, future research investigating brain-cardiac coupling within a monotonous or fatigued context should consider moving towards more realistic testing paradigms/conditions. A reasonable starting point would be to capture data either across or at multiple time points during the day/night cycle, as night driving has been associated with variations in markers of sleepiness and fatigue when compared to daytime driving [68,69,70]. Furthermore, electrophysiological parameters like EEG activity and HRV can be prone to circadian influence, and whilst this variance is accounted for in the present experimental design (by restricting the time point of data collection across all participants), it would be poignant for future research to track the relationship of EEG parameters and HRV over the course of day. Finally, even though simulated tasks cover many of the fundamental components of driving, they are inherently different, e.g., simulators make use of computer controlled or AI peers rather than true confederates. And so, future studies should consider the value of transitioning to the use of both controlled and “real” driving conditions and/or closed circuits for their driving tasks rather than the presently used lab-based simulator task.

## 5. Conclusions

The present research investigated the relationship between HRV and brain activity recorded during monotonous driving in train drivers, and coupled the two sets of electrophysiological parameters. The analyses found a number of associations between both low and high frequency HRV and EEG activity primarily in the delta and gamma frequency EEG bands. The coupling between these electrophysiological parameters provides some insight, not only into the direct cardiac impact of monotony experienced by train drivers, but also the subsequent longer term effects that train drivers may experience due to their prolonged and repeated exposure to a monotonous work environment. However, it is equally possible that these relationships and the related neural-cardiac coupling reflect combative tactics employed by drivers as they attempt to actively overcome fatigue and/or improve their engagement with the driving task. Finally, looking more broadly the interrelationship demonstrated between neural and cardiac biomarker monitoring could have major implications, subject to further investigations, as it relates to the routine use of fatigue or drowsiness monitoring systems.

## Figures and Tables

**Table 1:** Significant associations between electroencephalography variables and log transformed low frequency heart rate variability of train drivers.

Dependent Variable	Independent Variable	n	r	*p*
LFln	O_1_ − δ	34	0.43	0.04
	Fp_2_ − θ	46	−0.40	0.03
	Fp_1_ − α	45	−0.43	0.02
	FT_8_ − β	38	−0.58	<0.01
	P_4_ − γ	39	0.41	0.03
	O_1_ − γ	37	0.39	<0.05

Key: F = Frontal; Fp = Frontal Pole; LFln = log-transformed low frequency heart rate variability; n = Sample size; O = Occipital; P = Parietal; T = Temporal; α = Alpha; β = Beta; δ = Delta; γ = Gamma; θ = Theta.

**Table 2:** Regression analysis for log-transformed low frequency heart rate variability and electroencephalography variables in train drivers.

	R = 0.72; R^2^ = 0.52; AR^2^ = 0.42; df = 3, 14; F = 5.14; *p* = 0.01 *					
Variable	β	SE of β	B	SE of B	t	*p*
Intercept			6.72	0.34	20.00	<0.001 *
O_1_ − δ	−0.34	0.19	<−0.01	<0.01	−1.83	0.08
FT_8_ − β	−0.44	0.18	−0.05	0.02	−2.40	0.03 *
O_1_ − γ	0.39	0.19	0.13	0.06	2.09	0.05

Key: df = Degrees of freedom; F = Frontal; O = Occipital; SE = Standard Error; T = Temporal; β = Beta; δ = Delta; γ = Gamma; * = Statistical significance.

**Table 3:** Significant associations between electroencephalography variables and normalised low frequency heart rate variability of train drivers.

Dependent Variable	Independent Variable	*n*	r	*p*
LFn.u.	C_3_ − δ	42	0.38	0.04
	Fp_1_ − α	45	0.43	0.02
	P_4_ − β	36	−0.43	0.02
	P_4_ − γ	39	−0.43	0.02
	P_8_ − γ	40	−0.39	0.04
	O_1_ − γ	37	−0.46	0.02

Key: C = Central; Fp = Frontal Pole; LFn.u. = Normalised low frequency heart rate variability; n = Sample size; O = Occipital; P = Parietal; α = Alpha; β = Beta; δ = Delta; γ = Gamma.

**Table 4:** Regression analysis for normalised low frequency heart rate variability and electroencephalography variables in train drivers.

	R = 0.75; R^2^ = 0.57; AR^2^ = 0.52; df = 2, 20; F = 13.11; *p* < 0.001 *					
Variable	β	SE of β	B	SE of B	t	*p*
Intercept			73.61	2.56	28.74	<0.001 *
C_3_ − δ	0.31	0.15	0.12	0.06	2.11	<0.05 *
O_1_ − γ	−0.65	0.15	−2.05	0.46	−4.42	<0.001 *

Key: C = Central; df = Degrees of freedom; O = Occipital; SE = Standard Error; δ = Delta; γ = Gamma; * = Statistical significance.

**Table 5:** Significant associations between electroencephalography variables and log transformed high frequency heart rate variability of train drivers.

Dependent Variable	Independent Variable	*n*	r	*p*
HFln	FT_7_ − δ	46	−0.39	0.04
	C_3_ − δ	42	−0.36	<0.05
	Fp_1_ − α	45	−0.56	<0.01
	FT_8_ − β	38	−0.41	0.03
	P_4_ − γ	39	0.47	0.01
	P_8_ − γ	40	0.38	<0.05
	O_1_ − γ	37	0.47	0.01

Key: C = Central; F = Frontal; Fp = Frontal Pole; HFln = log-transformed high frequency heart rate variability; n = Sample size; O = Occipital; P = Parietal; T = Temporal; α = Alpha; β = Beta; δ = Delta; γ = Gamma.

**Table 6:** Regression analysis for log-transformed high frequency heart rate variability and electroencephalography variables in train drivers.

	R = 0.79; R^2^ = 0.62; AR^2^ = 0.55; df = 3, 17; F = 9.14; *p* < 0.001 *					
Variable	β	SE of β	B	SE of B	t	*p*
Intercept			5.40	0.43	12.62	<0.001 *
C_3_ − δ	−0.17	0.15	−0.01	0.01	−1.13	0.27
FT_8_ − β	−0.32	0.15	−0.06	0.03	−2.09	0.05
O_1_ − γ	0.67	0.15	0.27	0.06	4.40	<0.001 *

Key: C = Central; df = Degrees of freedom; F = Frontal; O = Occipital; SE = Standard Error; T = Temporal; β = Beta; δ = Delta; γ = Gamma; * = Statistical significance.

**Table 7:** Significant associations between electroencephalography variables and normalised high frequency heart rate variability of train drivers.

Dependent Variable	Independent Variable	*n*	r	*p*
HFn.u.	C_3_ − δ	42	−0.38	0.04
	Fp_1_ − α	45	−0.43	0.02
	P_4_ − β	36	0.43	0.03
	P_4_ − γ	39	0.43	0.02
	P_8_ − γ	40	0.38	0.04
	O_1_ − γ	37	0.46	0.02

Key: C = Central; Fp = Frontal Pole; HFn.u. = Normalised high frequency heart rate variability; n = Sample size; O = Occipital; P = Parietal; α = Alpha; β = Beta; δ = Delta; γ = Gamma.

**Table 8:** Regression analysis for normalised high frequency heart rate variability and electroencephalography variables in train drivers.

	R = 0.75; R^2^ = 0.56; AR^2^ = 0.52; df = 2, 20; F = 12.97; *p* < 0.001 *					
Variable	β	SE of β	B	SE of B	t	*p*
Intercept			26.34	2.55	10.32	<0.001 *
C_3_ − δ	−0.31	0.15	−0.12	0.06	−2.11	<0.05 *
O_1_ − γ	0.65	0.15	2.03	0.46	4.38	<0.001 *

Key: C = Central; df = Degrees of freedom; O = Occipital; SE = Standard Error; δ = Delta; γ = Gamma; * = Statistical significance.

**Table 9:** Significant associations between electroencephalography variables and log transformed total heart rate variability power of train drivers.

Dependent Variable	Independent Variable	*n*	r	*p*
TPowln	Fp_1_ − δ	45	−0.38	0.04
	Fp_2_ − θ	46	−0.39	0.03
	Fp_1_ − α	45	−0.47	0.01
	FT_8_ − β	38	−0.52	<0.01
	P_4_ − γ	39	0.46	0.01
	O_1_ − γ	37	0.46	0.02

Key: F = Frontal; Fp = Frontal Pole; n = Sample size; O = Occipital; P = Parietal; T = Temporal; TPowln = log-transformed total heart rate variability power; α = Alpha; β = Beta; δ = Delta; γ = Gamma; θ = Theta.

**Table 10:** Regression analysis for log-transformed total heart rate variability power and electroencephalography variables in train drivers.

	R = 0.81; R^2^ = 0.65; AR^2^ = 0.61;df = 2,17; F = 15.98; *p* < 0.001 *					
Variable	β	SE of β	B	SE of B	t	*p*
Intercept			930.22	851.69	1.09	0.29
P_4_ − γ	-0.20	0.14	−1830.53	1345.77	−1.36	0.19
O_1_ − γ	0.81	0.14	875.16	155.64	5.62	<0.001 *

Key: dfM = Degrees of freedom; O = Occipital; P = Parietal; SE = Standard Error; γ = Gamma; * = Statistical significance.

**Table 11:** Significant associations between electroencephalography variables and the low/high frequency heart rate variability ratio of train drivers.

Dependent Variable	Independent Variable	*n*	r	*p*
LF/HF	FT_7_ − δ	46	0.53	<0.01
	FT_8_ − δ	46	0.48	<0.01
	FT_7_ − θ	47	0.39	0.04
	FC_4_ − θ	40	0.63	<0.001
	Fp_1_ − α	45	0.52	<0.01
	CP_z_ − β	40	0.49	0.01
	FC_3_ − γ	35	0.54	<0.01
	CP_z_ − γ	40	0.45	0.02

Key: C = Central; F = Frontal; Fp = Frontal Pole; HF = High-frequency heart rate variability; LF = Low-frequency heart rate variability; n = Sample size; O = Occipital; P = Parietal; T = Temporal; z = Midline; α = Alpha; β = Beta; δ = Delta; γ = Gamma; θ = Theta.

**Table 12:** Regression analysis for the low/high frequency heart rate variability ratio and electroencephalography variables in train drivers.

	R = 0.78; R^2^ = 0.61; AR^2^ = 0.56;df= 2, 17; F = 13.07; *p* < 0.001 *					
Variable	β	SE of β	B	SE of B	t	*p*
Intercept			1.59	0.83	1.92	0.07
FT_8_ − δ	0.59	0.22	0.03	0.01	2.61	0.02 *
FC_4_ − θ	0.24	0.22	0.13	0.12	1.06	0.30

Key: C = Central; df = Degrees of freedom; F = Frontal; O = Occipital; T = Temporal; δ = Delta; θ = Theta; * = Statistical significance.

## Data Availability

The data relevant to the current study are available from the corresponding author on reasonable request.
